# CDC Recommendations for Hepatitis C Testing Among Perinatally Exposed Infants and Children — United States, 2023

**DOI:** 10.15585/mmwr.rr7204a1

**Published:** 2023-11-03

**Authors:** Lakshmi Panagiotakopoulos, Amy L Sandul, DHSc, Erin E. Conners, Monique A. Foster, Noele P. Nelson, Carolyn Wester, Collaborators, Collaborators, Elizabeth Barnett, Ravi Jhaveri, Gwen Lazenby, Christine Lee, Wael Mourad, Adam Ratner

**Affiliations:** ^1^Division of Viral Hepatitis, National Center for HIV, Viral Hepatitis, STD, and TB prevention, CDC; ^2^Division of Global Health Protection, Center for Global Health, CDC; Boston University Chobanian & Avedisian School of Medicine; Ann & Robert H. Lurie Children’s Hospital of Chicago; Medical University of South Carolina; Harvard Medical School; University of Missouri–Kansas City School of Medicine; Hassenfeld Children’s Hospital.

## Abstract

*The elimination of hepatitis C is a national priority *(https://www.hhs.gov/sites/default/files/Viral-Hepatitis-National-Strategic-Plan-2021-2025.pdf)*. During 2010–2021, hepatitis C virus (HCV) acute and chronic infections (hereinafter referred to as HCV infections) increased in the United States, consequences of which include cirrhosis, liver cancer, and death. Rates of acute infections more than tripled among reproductive-aged persons during this time (from 0.8 to 2.5 per 100,000 population among persons aged 20–29 years and from 0.6 to 3.5 among persons aged 30–39 years). Because acute HCV infection can lead to chronic infection, this has resulted in increasing rates of HCV infections during pregnancy. Approximately 6%–7% of perinatally exposed (i.e., exposed during pregnancy or delivery) infants and children will acquire HCV infection. Curative direct-acting antiviral therapy is approved by the Food and Drug Administration for persons aged ≥3 years. However, many perinatally infected children are not tested or linked to care. In 2020, because of continued increases in HCV infections in the United States, CDC released universal screening recommendations for adults, which included recommendations for screening for pregnant persons during each pregnancy* (Schillie S, Wester C, Osborne M, Wesolowski L, Ryerson AB. CDC recommendations for hepatitis C screening among adults—United States, 2020. MMWR Recomm Rep 2020;69[No. RR-2]:1–17). *This report introduces four new CDC recommendations: 1) HCV testing of all perinatally exposed infants with a nucleic acid test (NAT) for detection of HCV RNA at age 2–6 months; 2) consultation with a health care provider with expertise in pediatric hepatitis C management for all infants and children with detectable HCV RNA;* *3)* *perinatally exposed infants and children with an undetectable HCV RNA result at or after age 2 months do not require further follow-up unless clinically warranted; and 4) a NAT for HCV RNA is recommended for perinatally exposed infants and children aged 7–17 months who previously have not been tested, and a hepatitis C virus antibody (anti-HCV) test followed by a reflex NAT for HCV RNA (when anti-HCV is reactive) is recommended for perinatally exposed children aged ≥18 months who previously have not been tested. Proper identification of perinatally infected children, referral to care, and curative treatment are critical to achieving the goal of hepatitis C elimination.*

## Introduction

Hepatitis C virus (HCV) is a single-stranded RNA virus that causes liver inflammation that can progress over time to advanced fibrosis, cirrhosis, and hepatocellular carcinoma (HCC) (*1*–*3*). Rates of HCV acute and chronic infections (referred hereinafter as HCV infections) have been steadily increasing in the United States since 2010, with rates of acute infections more than tripling among reproductive-aged persons as of 2021, from 0.8 to 2.5 per 100,000 population among persons aged 20–29 years and from 0.6 to 3.5 among persons aged 30–39 years (*4*,*5*). As a result of increasing rates of acute infections in reproductive-aged persons and subsequent chronic infections, overall rates of HCV infections during pregnancy have increased by 20% during 2016–2020 and up to tenfold during 2000–2019 (*6*,*7*). HCV is transmitted through percutaneous exposure to infected blood; increases in infection rates have corresponded to increases in injection drug use(IDU) (*8*–*11*). In 2020, because of the changing epidemiology of HCV infections in the United States, CDC expanded previous risk-based testing recommendations to include universal screening for all adults aged ≥18 years at least once and for all pregnant persons during each pregnancy (*12*). Studies have estimated that chronic HCV infection will develop in 5.8%–7.2% of all perinatally exposed (i.e., exposed during pregnancy or delivery) infants and children (*13*,*14*), and curative direct-acting antiviral (DAA) therapy can be administered beginning at age 3 years (*15*,*16*). However, most perinatally exposed infants and children are not tested for HCV infection and are not referred for hepatitis C care (*17*–*20*); reasons for this might include lack of awareness of perinatal exposure by pediatric providers, lack of regular pediatric care among exposed infants and children, changes in health care providers before the time of HCV testing (recommended at age 18 months), and challenging social circumstances for parents and guardians.

This report supplements the 2020 CDC recommendations for HCV screening among adults in the United States (*12*), which includes universal screening among pregnant persons during each pregnancy, by recommending the timing and type of test for diagnosis of current HCV infection for infants and children born to HCV-infected pregnant persons. Because HCV epidemiology and methods of testing infants and children for HCV infection have evolved, this report replaces a previous recommendation for testing perinatally exposed infants and children included in a CDC recommendation from 1998 (*21*). This report is intended to serve as a resource for persons involved in the development, implementation, delivery, and evaluation of clinical and preventive services, including health care professionals; public health officials; and professional, academic, and public health and advocacy organizations. If recommendations are implemented, more perinatally infected children will be identified and linked to care. This approach would increase the chances of timely treatment and subsequent cure that can mitigate the consequences from chronic hepatitis C and limit further transmission.

## Hepatitis C Epidemiology, Transmission, Diagnosis, and Management

### Hepatitis C Epidemiology

**FIGURE 1:**
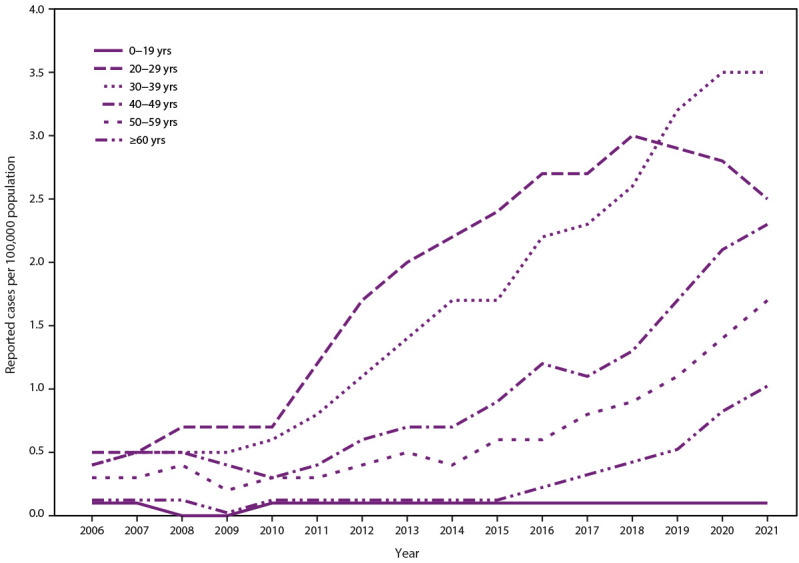
Rates* of laboratory confirmed acute hepatitis C virus infection,^†^ by age group — United States, 2006–2021^§,¶^ **Source:** National Notifiable Diseases Surveillance System, CDC. * Rates per 100,000 population. ^†^ Reported confirmed cases. For the case definition, see https://ndc.services.cdc.gov/conditions/hepatitis-c-acute/. ^§^ The number of viral hepatitis cases reported to CDC in 2020 and 2021 might be lower than in years before the COVID-19 pandemic began. The decrease might be related to fewer people seeking health care and being tested for viral hepatitis during the COVID-19 pandemic. ^¶^ Changes in case definitions should be considered when examining temporal trends. For more information regarding the case definitions used in 2021, see https://www.cdc.gov/nndss/index.html.

**FIGURE 2:**
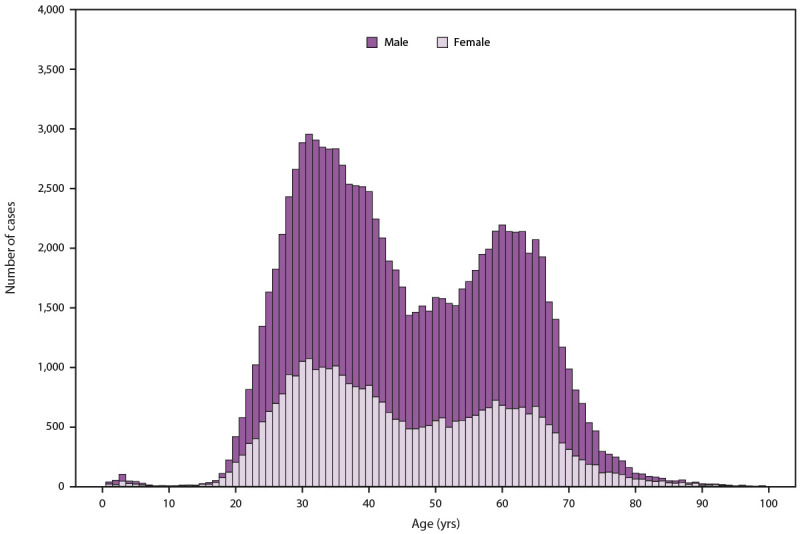
Number of laboratory confirmed* chronic hepatitis C virus infection cases,^†^ by sex and age — United States, 2021^§,¶^ **Source:** National Notifiable Diseases Surveillance System, CDC. * During 2021, cases of chronic hepatitis C were either not reportable by law, statute, or regulation; not reported; or unavailable to CDC from Arizona, District of Columbia, Hawaii, Indiana, Kentucky, North Carolina, Rhode Island, and Texas. ^†^ Only laboratory confirmed, newly diagnosed, chronic hepatitis C cases are included. For the case definition, see https://ndc.services.cdc.gov/conditions/hepatitis-c-chronic/. ^§^ The number of viral hepatitis cases reported to CDC in 2020 and 2021 might be lower than in years before the COVID-19 pandemic began. The decrease might be related to fewer people seeking health care and being tested for viral hepatitis during the COVID-19 pandemic. ^¶^ Changes in case definitions should be considered when examining temporal trends. For more information regarding the case definitions used in 2021, see https://www.cdc.gov/nndss/index.html.

In 2021, a total of 5,023 cases of acute hepatitis C were reported to CDC, a rate of 1.6 cases per 100,000 persons (*4*). However, because underreporting and underascertainment are common, CDC estimated that approximately 69,800 acute infections occurred during 2021 (95% CI = 55,300–238,100) (*22*). Rates have increased annually since 2010 and were highest among persons aged 20–39 years, representing approximately 52% of reported acute cases (Figure 1). These increasing rates also occurred among groups at highest risk for fatal overdose from IDU (*10*). Although data on risk behaviors or exposures are most often missing, among those cases with risk factors reported, approximately 57% reported IDU. Furthermore, because of the increase in acute HCV infections, newly reported chronic cases are now highest among persons aged 20–39 years (Figure 2). Additional information on the epidemiology of reported acute and chronic hepatitis C cases is available at https://www.cdc.gov/hepatitis/statistics/2021surveillance/hepatitis-c.htm.

Although a history of IDU is the most commonly reported risk factor among adults for acquiring HCV infection, perinatal transmission is the primary risk factor among young children. Perinatal HCV infection has been notifiable since 2018 (*23*). In 2021, a total of 199 perinatal hepatitis C cases were reported to CDC from 28 states, with approximately one half of cases reported by six states; this approximation is likely a substantial underestimate of the number of cases in the United States (*24*). The states with the highest number of cases were Ohio (42), Pennsylvania (15), Indiana (15), and Tennessee (12) (*4*). A study using commercial laboratory data during 2011–2014 determined that 0.73% (95% CI = 0.71%–0.75%) of pregnant persons tested had current HCV infection (*25*). Applying these proportions to the annual birth rate during 2011–2014, the study estimated that 29,000 women with HCV infection gave birth to 1,700 infants with perinatal HCV infection each year.

### Virology and Transmission of HCV

HCV, previously known as posttransfusion non-A, non-B hepatitis virus, was first characterized in 1989 (*26*,*27*). HCV is a single-stranded RNA virus belonging to the Flaviviridae family and includes at least seven genotypes known to infect humans, with differences in geographic spread and implications for treatment (*28*–*33*). Approximately 90% of infections globally represent genotypes 1, 2, 3, and 4; in the United States, genotype 1 is most common (*31*).

HCV is a bloodborne pathogen spread parenterally; the most common mode of transmission is IDU. HCV infection also can be transmitted to the fetus and newborn during pregnancy and delivery, respectively. Less common modes of transmission include sexual contact, health care procedures, needlestick injuries in health care settings, unregulated tattooing, and sharing personal items contaminated with infectious blood. Historically, children were most commonly infected through transfusions; however, routine screening of blood products since 1992 has largely eliminated this mode of transmission in the United States (*4*,*34*,*35*).

#### Perinatal HCV Transmission

A systematic review and meta-analysis of 109 articles found that the risk for perinatally acquired infection from an HCV antibody-reactive pregnant person with detectable HCV RNA was 5.8% (95% CI = 4.2%–7.8%) in the absence of HIV infection and 10.8% (95% CI = 7.6%–15.2%) among those with poorly controlled HIV coinfection (*13*). A statistical reanalysis of data, including 1,749 children in three prospective cohorts that corrected for infections that might have cleared before they were detected, calculated an overall perinatal transmission rate of 7.2% (95% CI = 5.6%–8.9%) among pregnant persons who were HIV negative and 12.1% (95% CI = 8.6%–16.8%) among those with poorly controlled HIV coinfection (*14*). This study also estimated that 24.8% of perinatal infections occur early in utero, 66% later in utero, and 9.3% during delivery.

Perinatal transmission is limited to infants born to pregnant persons with detectable HCV RNA (*36*,*37*). Transmission occurs more frequently among pregnant persons with poorly controlled HIV coinfection and also might be more common in pregnant persons injecting drugs (*36*,*38*–*42*). The use of antiretroviral therapy for HIV during pregnancy to lower HIV viral load is associated with lower rates of perinatal HCV transmission, which are closer to transmission rates among pregnant persons with HCV monoinfection (*43*–*45*). Although detectable HCV RNA in a pregnant person is a known risk factor for perinatal transmission, the specific levels of HCV RNA or a specific HCV genotype are not known to be associated with increased risk for transmission (*36*,*38*). Giving birth to a child who was perinatally infected also does not increase risk for perinatal transmission over baseline in subsequent pregnancies; HCV infections are equally distributed among first-born infants and subsequent births (*46*). Data on prenatal testing and risk for perinatal HCV transmission are limited; however, amniocentesis is usually recommended over chorionic villus sampling when invasive testing is indicated (*47*–*49*). Caesarean delivery is not recommended over vaginal delivery to prevent perinatal HCV transmission (*36*,*50*). Membrane rupture lasting ≥6 hours before delivery and the use of internal fetal monitoring have been associated with an increased risk for transmission (*37*). After delivery, breastfeeding does not increase HCV transmission unless nipples are cracked or bleeding (*36*,*48*).

### Impact of Maternal HCV Infection on Pregnancy and Neonatal Outcomes

Data on the effects of HCV infection on pregnancy, birth, and neonatal outcomes have been mixed (*51*–*57*). Associations might be confounded by maternal substance use during pregnancy and unmeasured sociodemographic factors. However, studies have consistently illustrated an increased risk for gestational diabetes mellitus and intrahepatic cholestasis of pregnancy to be associated with HCV infection (*52*,*54*,*56*,*58*). Certain studies have found associations between HCV infection and adverse birth and neonatal outcomes. A meta-analysis of birth outcomes after HCV infection among 4,185,414 participants, 5,094 of whom had HCV infection, found a statistically significant association between maternal HCV infection and intrauterine growth restriction and low birthweight (*55*). A population-based cohort study in Washington using birth certificate data compared 506 pregnant persons infected with HCV with 2,022 mothers who were HCV negative and 1,439 mothers who were HCV negative and known to use drugs (*52*). After controlling for maternal age, race, tobacco use, alcohol use, and prenatal care, infants perinatally exposed to HCV infection were more likely to have low birthweight, be small for gestational age, and have neonatal intensive care unit (NICU) admission and assisted ventilation. In addition, compared with the maternal drug-using cohort without HCV infection, the maternal cohort with HCV infection was more likely to have infants with NICU admission who required assisted ventilation. A study of births in Florida determined that after adjusting for sociodemographic variables and obstetric complications, HCV infection during pregnancy was associated with preterm delivery, low birthweight infants, and congenital anomalies (*54*). Another study indicated approximately 22% increased odds of having an infant with an adverse neurologic outcome (odds ratio = 1.22; 95% CI = 1.03–1.44) among pregnant persons with HCV infection compared with pregnant persons without HCV infection (*59*).

### Clinical Features and Natural History of Perinatally Acquired HCV Infection

Among children with perinatally acquired HCV infection, spontaneous clearance of infection (i.e., resolution of the infection without treatment resulting in undetectable virus) typically occurs in 20%–40% of children by age 5 years (*37*,*60*–*64*). However, a recent study analyzed data from three prospective studies, accounting for interval censoring and left-truncated data, to evaluate HCV RNA clearance more precisely by age (*65*). Among 106 infants aged <36 months with current infection included in the analysis, 57.3% cleared by age 3 years and 65.9% cleared by age 5 years. Clearance is associated with sustained undetectable HCV RNA; viral RNA levels are initially high and then slowly decline. Antibody to hepatitis C virus (anti-HCV) typically persists for life but can wane over time (*62*).

Limited studies have evaluated long-term outcomes among children perinatally infected with HCV (*64*,*66*). One of the largest studies was a cohort of 266 children infected perinatally and followed prospectively through a median age of 4.2 years (range: 3.2 months to 15.9 years) (*64*). Children were recruited from 30 European Paediatric HCV Network centers and were considered infected if they had ≥2 positive nucleic acid tests (NATs) for HCV RNA or a positive anti-HCV test at age ≥18 months. In this study, 10% of children had hepatomegaly, which was found to be associated with elevated alanine aminotransaminase (ALT) levels. Among the children with hepatomegaly, the study estimated median age at first diagnosis to be 7.1 months, with approximately 13% of children developing hepatomegaly by age 5 years and approximately 28% by age 10 years. Among the infected cohort, approximately 20% of children cleared the infection (median age: 14.9 months), 50% had chronic asymptomatic infection, and 30% had chronic active infection (i.e., persistent viremia with or without hepatomegaly and elevated ALT). Another prospective study of 45 children infected perinatally found that all children were asymptomatic for liver disease; however, 11 children had evidence of mild-to-moderate fibrosis (*62*). In addition, autoimmune phenomena, including nonorgan-specific autoantibodies, cryoglobulinemia, low C4 levels, and persistent proteinuria were also found among perinatally infected children.

A retrospective study followed 35 perinatally infected children recruited from seven European centers for a mean of 4 years (SD: 2.2 years) after diagnosis of HCV infection (*67*). Of these children, 77% had persistently or intermittently abnormal ALT levels. In addition, ALT levels tended to be higher during the first year of life among those in the study who subsequently spontaneously cleared their infections. Another prospective study of 45 children with perinatally acquired HCV infection (median age: 12 years) found that children who spontaneously cleared the infection had higher ALT levels during the first 2 years of life than those who did not (*62*).

Descriptive evaluations of liver pathology among children with chronic hepatitis C have been reported (*68*–*70*). However, clinical outcomes in these studies are not generalizable to all children at risk because findings are reported among a sample of children with chronic hepatitis C with clinical indications for liver biopsy, including severe progression of disease. One report of 121 treatment-naïve children aged 2–16 years indicated that inflammation on liver biopsy was minimal in approximately 42% of children, mild in 17%, moderate in 38%, and severe in 3% (*68*). Among these 121 patients, five had bridging fibrosis and two had cirrhosis; degree of liver inflammation correlated with duration of infection. Another study of 60 HCV-infected children, including 13% who were perinatally infected, found that 71% had mild-to-minimal inflammation on liver biopsy, and 88% had absent or minimal fibrosis (*69*). However, in this study, three of eight children infected perinatally had cirrhosis, one of whom had slow progression and two of whom required liver transplantation and subsequently died from transplant complications. One study evaluated the progression of histologic liver disease by comparing initial and repeat liver biopsies (mean of 5.8 years; SD: 3.5 years) among 44 children with chronic hepatitis C, 57% of whom were infected perinatally (*70*). Among 25 children infected perinatally, 12% had no fibrosis, 76% had portal or periportal fibrosis, and 12% had bridging fibrosis or cirrhosis. Although a statistically significant progression of histologic liver disease among the aggregate cohort was not found, a statistically significant increase in the severity of fibrosis in 30% of children was observed.

### Diagnosis of HCV Infection Among Pregnant Persons and Perinatally Infected Children

Hepatitis C screening with an anti-HCV test is recommended for all pregnant persons during each pregnancy (*12*,*71*). A nonreactive test (i.e., no detectable antibody or negative antibody) indicates no HCV antibody detected and the person likely has never been infected. A nonreactive anti-HCV result also might represent an acute infection during the window period (if exposure was recent) or, among rare cases, a seronegative infection. A reactive HCV antibody (i.e., detectable antibody or positive antibody) indicates current or past infection and should be followed with a NAT for HCV RNA (i.e., reflex NAT for HCV RNA). An undetectable HCV RNA result by NAT indicates no current HCV infection. A detectable HCV RNA result is indicative of current HCV infection, and the person should be linked to care and considered for treatment.

Before 2018, recommendations for hepatitis C testing during pregnancy were limited to persons with known risk factors (*12*). However, this strategy left multiple infections undiagnosed (*72*). In 2018, because of low testing rates and increases in HCV infections among persons of reproductive age, the Infectious Diseases Society of America (IDSA) and the American Association for the Study of Liver Diseases (AASLD) recommended universal screening during pregnancy (*48*). In 2020, both the U.S. Preventive Services Task Force (USPSTF) and CDC recommended universal screening during pregnancy (*12*,*73*). This universal screening during pregnancy recommendation was followed by similar recommendations from two obstetrical organizations, the American College of Obstetrics and Gynecology (ACOG) and the Society of Maternal Fetal Medicine (SMFM) in 2021 (*49*,*74*).

Because recommendations have recently shifted from risk-based to universal screening, uptake of HCV screening during pregnancy is evolving, as demonstrated by two studies using commercial laboratory data. During 2011–2016, analysis of laboratory data determined HCV testing increased from 5.7% to 13.4% among pregnant persons within the population eligible for inclusion in the study (*75*). Another analysis of 5,048,428 pregnant persons using laboratory data indicated an increase in the percentage of pregnant persons with an HCV screening test from 16.6% in 2011 to 40.6% in the second quarter of 2021 (*76*). Systematic review of the literature indicated the median anti-HCV prevalence during pregnancy was 1.2% (range: 0.1%–70.8%); 66.1% (range: 61.3%–77.2%) of pregnant persons with a reactive anti-HCV test also had detectable HCV RNA (*4*,*12*).

All professional societies with perinatal hepatitis C testing guidelines recommend testing perinatally exposed infants aged ≥18 months with an HCV antibody test (*3*,*15*,*77*,*78*). Anti-HCV tests should not be performed earlier than age 18 months in perinatally exposed children because of passive transfer of maternal antibody. NAT for HCV RNA can be done as early as age 2 months in perinatally exposed infants, which might decrease loss to follow-up and help alleviate anxiety among families wanting to know an infant’s HCV infection status (*17*–*19*,*77*). NAT for HCV RNA cannot be used before age 2 months for diagnosis of perinatal HCV transmission because of false-negative results. Furthermore, the presence of HCV RNA during the first weeks of life might indicate contamination with maternal blood or passive transfer of maternal HCV RNA rather than newly established HCV infection of the infant (*37*). Authors from a single center retrospectively reviewed more than 10 years of laboratory and clinical data for all infants who had perinatal HCV exposure (*79*) and found that nearly all those who had undetectable HCV RNA at age 2–6 months were HCV seronegative at 24 months (223 of 226; 98.7%), and all 226 infants were clinically classified as true negatives for HCV infection. In this study, the sensitivity of a single NAT for HCV RNA at age 2–6 months was 100% (95% CI = 87.5%–100.0%), and the specificity was 100% (95% CI = 98.3%–100.0%). Furthermore, using the perinatal transmission rate of 3.6% calculated in the study, the positive-predictive value (PPV) of a single NAT for HCV RNA was 100% (95% CI = 74.5.%–100.0%), and the negative-predictive value (NPV) was 100% (95% CI = 99.6%–100.0%). Using a perinatal transmission rate of 5.8% from the literature, the PPV of a single NAT for HCV RNA was 100% (95% CI = 82.8%–100.0%), and the NPV was 100% (95% CI = 99.4%–100.0%). More recent estimates indicate a perinatal transmission rate closer to 7% (*14*), which would be expected to increase the lower bound of the PPV CI. Standalone NATs for HCV RNA detection are not FDA approved for diagnosis of HCV infection; off-label use of an FDA-approved diagnostic test requires validation by the testing laboratory. Because spontaneous clearance occurs among up to 65.9% of children by age 5 years (*37*,*60*–*65*), the American Association for the Study of Liver Diseases and the Infectious Diseases Society of America (AASLD-IDSA) guidelines recommend a NAT for HCV RNA to confirm current infection before initiation of DAA therapy, which can be started at age 3 years.

### Clinical Management and Treatment of Perinatal HCV Infection

Approximately 6%–7% of perinatally exposed children will acquire perinatal HCV infection. Curative DAA therapy is FDA approved for children aged ≥3 years; however, more than one half of perinatally infected children are not tested or linked to care.

CDC assessed evidence in support of recommending perinatal testing for hepatitis C among infants to maximize early diagnosis and linkage to care and increase the number of children who receive curative treatment before developing clinical manifestations and complications from chronic hepatitis C. Standard practice for clinical management and treatment of perinatal HCV infection is outside the scope of this CDC recommendation. AASLD-IDSA and the North American Society for Pediatric Gastroenterology, Hepatology, and Nutrition (NASPGHAN) have published guidelines for the clinical management and treatment of perinatal HCV infection (*15*,*16*,*77*).

## Methods

The Recommendations Steering Committee, composed of CDC staff with expertise in viral hepatitis, obstetrics, pediatrics, infectious diseases, and policy (Supplementary Appendix 1, https://stacks.cdc.gov/view/cdc/134020), met regularly to oversee the development of the recommendations. The steering committee designed a comprehensive systematic review of the literature to guide the decision for these recommendations. The purpose of the review was to examine the benefits and harms of different testing strategies for identifying children with perinatally acquired HCV infection.

**TABLE 1:** Median and range of number of studies in literature review related to perinatal hepatitis C testing, prevalence, and linkage to care, by selected characteristics — United States, 2023

Characteristic	Median % (IQR)	Range, %	No. of studies guiding estimates
Pregnant persons*			
Proportion tested for HCV	47.6 (10.2–80.9)	0.7–98.4	16
Prevalence of reactive anti-HCV or diagnosis	1.1 (0.4–30.9)	0.1–70.8	35
Prevalence of detectable HCV RNA^†^	68.2 (61.5–77.2)	29.6–81.3	11
**Perinatally exposed children**			
Proportion referred for HCV testing	16.7 (NA)^§^	1.9–31.4	2
Proportion tested for HCV^¶^	30.1 (20.3–44.5)	8.6–53.1	12
Rate of perinatal transmission	4.7 (3.2–7.4)	0.0–11.1	13
Proportion of infected children linked to care**	100% (NA)	NA	1
Proportion of children with CHC who achieved SVR12 after DAA treatment	98.1 (97.1–98.9)	96-100	7

**Abbreviations:** anti-HCV = hepatitis C virus antibody; CHC = chronic hepatitis C; DAA = direct-acting antiviral; HCV = hepatitis C virus; IQR = interquartile range; NA = not available; NAT = nucleic acid test; SVR12 = sustained virologic response 12 weeks posttreatment.

* All estimates among pregnant persons include studies reviewed in **Source:** Schillie S, Wester C, Osborne M, Wesolowski L, Ryerson AB. CDC recommendations for hepatitis C screening among adults—United States, 2020. MMWR Recomm Rep 2020;69:1–17.

^†^ HCV RNA detected among those who are anti-HCV reactive.

^§^ No IQR available because only two studies.

^¶^ Perinatally exposed children tested with an anti-HCV test or a NAT for HCV RNA.

** Children who received a diagnosis of perinatal hepatitis C who attended an HCV-related appointment.

The following research question guided the development of the recommendations:

- Among children perinatally exposed to HCV, does NAT for HCV RNA at age 2–6 months^*^compared with HCV antibody testing with reflex RNA testing (i.e., NAT for HCV RNA after a reactive anti-HCV test) at age ≥18 months, increase the diagnosis of current HCV infections, increase linkage to care and treatment, and decrease cirrhosis and deaths attributable to HCV infection?

This question was further broken down into five questions guiding a chain of indirect evidence:

- Compared with HCV antibody testing at age ≥18 months, how would NAT for HCV RNA at age 2–6 months affect the number of children identified with perinatally acquired HCV infection?
- How many additional children with perinatally acquired HCV infection would be identified by testing with NAT for HCV RNA at age 2–6 months?
- How many additional children with perinatally acquired HCV infection would be linked to care by testing with NAT for HCV RNA at age 2–6 months?
- Do desirable effects (i.e., benefits) of testing for HCV infection outweigh undesirable effects (harms)?
- What is the effect of diagnosis at age 2–6 months with NAT for HCV RNA on cirrhosis and deaths attributable to HCV infection?

Key questions (KQs) were developed for each of the five questions from the chain (Supplementary Table 1, https://stacks.cdc.gov/view/cdc/133599):

- K.Q.1.a. What is the prevalence of HCV infection among pregnant persons in the United States?
- K.Q.1.b. What proportion of pregnant persons are tested for HCV infection in the United States?
- K.Q.1.c. What proportion of children perinatally exposed to HCV become infected?
- K.Q.2.a. What is the diagnostic accuracy of HCV antibody testing and NAT for HCV RNA among perinatally exposed children?
- K.Q.2.b. What proportion of children perinatally exposed to HCV are tested for HCV infection?
- K.Q.3.a. What proportion of children with confirmed HCV infection are linked to care?
- K.Q.4.a. What are the benefits of HCV testing among perinatally exposed children?
- K.Q.4.b. What are the harms of HCV testing among perinatally exposed children?
- K.Q.5.a. What is the effect of hepatitis C diagnosis in childhood on related morbidity and mortality (including cirrhosis, HCC, and death)?
- K.Q.5.b. What is the effect of DAA treatment in childhood on hepatitis C–related morbidity (including cirrhosis and HCC)?

### Literature Review

A systematic review of the literature was conducted to examine available evidence on HCV infection prevalence among pregnant persons and perinatally exposed children, loss of follow-up among perinatally exposed children, and the benefits and harms of testing perinatally exposed children.

A search for English language, peer-reviewed journal articles published in Medline (Ovid), Embase (Ovid), Cochrane Library, CINAHL (EBSCO), and Scopus was performed (Supplementary Table 2, https://stacks.cdc.gov/view/cdc/133599). The search included articles published during January 1, 2001–June 8, 2021. The 20-year period was selected because of the expected scarcity of data among this population. Duplicates were identified using EndNote software (version 20; Clarivate), which automated the “find duplicates” function with preference set to match on title, author, and year. Duplicates were removed from the EndNote library.

All references from the initial search were uploaded into DistillerSR software (version 2.35; Evidence Partners) for further review by the Recommendations Work Group (Supplementary Appendix 1, https://stacks.cdc.gov/view/cdc/134020). Two independent reviewers checked all titles and abstracts for relevance to the research question (AS, JB, or NN and LP). Titles determined to be non-English language articles or not relevant to the study question were not included in the abstract review. All articles determined to be relevant in the title review and articles with conflicting results in the title review were included in the abstract review. Similarly, all abstracts determined to be relevant to the research question and articles with conflicting inclusion results in the abstract review were included for full text review.

Included articles were separated into three categories for the full text review: 1) U.S. articles only discussing HCV in pregnancy (without data on perinatal HCV), 2) U.S. articles that included data regarding perinatal HCV testing, and 3) international articles that potentially included harms of perinatal HCV testing. All full text articles were independently reviewed by two reviewers (AS, EC, JB, LC, or MF and LP). Relevant data were abstracted independently and compared. All differences in abstracted data were discussed by the two reviewers until they reached agreement.

Articles were excluded if an English language version could not be found, were not related to HCV infection, treatment, or testing; were international articles not specific to perinatal HCV transmission and testing; were case reports, case series, opinion articles, editorials, review articles, or conference abstracts; contained only modeled data or only animal data; included adults aged ≥18 years (unless also included perinatally infected or pregnant persons); reported HCV infection among children not acquired perinatally; or only reported on medications not recommended for use among children. Data on testing rates and prevalence of HCV infection during pregnancy that had been considered in the development of the 2020 report on CDC recommendations for HCV screening among adults were also included in this literature summary (*12*). On completion of the formal literature review, reference lists from all U.S. and international review articles were reviewed to identify additional articles for full text review.

After data abstraction, all included articles related to pregnancy and perinatal HCV testing rates, incidence, linkage to care, and outcomes underwent review to assess the quality of the evidence using the National Institutes of Health (NIH) Study Quality Assessment Tools, which were developed specific to certain study designs and focus on concepts key to a study’s internal validity (Supplementary Table 3, https://stacks.cdc.gov/view/cdc/133599). Articles were first categorized by study design, and each criterion and overall rating was independently scored by two reviewers (AS or EC and LP) using the descriptions available at https://www.nhlbi.nih.gov/health-topics/study-quality-assessment-tools. The two reviewers discussed and reached agreement on giving the articles an overall rating of good, fair, or poor. All articles, regardless of their rating, were included in the overall analysis. Because articles describing harms of perinatal HCV screening varied in the relation between the study design and the harm mentioned, the quality of U.S. and international articles on the harms of perinatal HCV testing was scored based on two standardized measures developed by the Recommendations Steering Committee: 1) level of confidence (high, moderate, or low), indicating how the specific harm was measured in the study, and 2) outcome prioritization (critical, important, not important), indicating the relevance of the harm as it related to the study question. Although these determinations were based on expert opinion and judgment, two reviewers (AS, EC, or MF and LP) independently scored each study with a harm; discrepancies in the level of confidence or the outcome prioritization were discussed by the two reviewers until they reached agreement. The quality assessment of the cost-effectiveness study was evaluated by two independent reviewers (TA and LP) using the Consolidated Health Economic Evaluation Reporting Standards (CHEERS) checklist (*80*).

To identify recently published studies through December 31, 2022, a supplemental literature search was conducted on January 17, 2023, using a search strategy identical to the original search (Supplementary Table 4, https://stacks.cdc.gov/view/cdc/133599). Titles and abstracts were independently reviewed by NN or MW and LP. All studies with conflicting agreement on inclusion proceeded for full text reviews, which were independently reviewed by AS or EC and LP. Evidence quality reviews for all included articles were conducted using the same methods as for the original reviews by AS or EC and LP. Abstracted data and evidence assessments from full text reviews were added to the original review.

In developing recommendations, the Recommendations Steering Committee considered the results of the literature review and findings from the effectiveness and cost-effectiveness modeling study (see Evidence Summary). Furthermore, the steering committee had biweekly meetings to discuss implementation feasibility, public health implications, and equitable access to testing.

CDC determined that these recommendations included influential scientific information with a clear and substantial impact on important public policies and private sector decisions. As required by the Information Quality Act (*81*), peer review by external specialists not involved in the development of the recommendations was conducted. CDC solicited nominations for peer reviewers from the American Academy of Pediatrics (AAP), NASPGHAN, AASLD, the American Academy of Family Physicians (AAFP), and ACOG. Six peer reviewers from the listed organizations with expertise in pediatrics, infectious diseases, hepatology, and obstetrics reviewed the recommendations and provided structured peer reviews (Supplementary Appendix 1, https://stacks.cdc.gov/view/cdc/134020). Representatives from professional societies, providers, advocacy groups, and public health professionals have communicated the need for clear recommendations through conferences, journal commentary, and other types of communications. Buy-in from relevant parties was obtained at inception of recommendation development in April 2021, and methods for developing the recommendations were summarized in a presentation only (i.e., no vote was conducted) at the April 2022 meeting of the CDC/Health Resources and Services Administration Advisory Committee on HIV, Viral Hepatitis and STD Prevention and Treatment. Opportunity for reaction and feedback to the draft recommendation was provided through a public comment period (November 22, 2022–January 27, 2023) and an informational webinar open to the public, academia, advocacy groups, and partner organizations. CDC received 22 public comments on the draft document from the public, providers, advocacy groups, industry, medical professional associations, think tanks, and one public health department. Peer reviewer and public comments were considered by the work group, and edits made in response were documented (Supplementary Appendix 2 and Supplementary Appendix 3, https://stacks.cdc.gov/view/cdc/134020).

### Summary of the Literature

The initial literature search yielded 3,802 articles. All titles were screened, 1,241 (32.6%) abstracts were reviewed, and 201 (5.3%) full texts were reviewed for possible inclusion. A total of 35 articles (0.9%) from the initial literature review had data available to abstract. An additional six articles were identified from references and were included for data abstraction. After review, 41 articles were included.

The supplementary literature search yielded an additional 459 articles. All titles were screened, 160 abstracts (34.9%) were reviewed, and 23 (5.0%) full texts were reviewed for possible inclusion. A total of 11 articles (2.4%) from the supplementary literature review had data to abstract. An additional four articles were identified from references and were included for abstraction. The supplementary review added 15 articles to the original 41 for a total of 56 articles included.

Sixteen articles included data related to HCV testing rates during pregnancy, 12 of which were reviewed in the 2020 CDC adult HCV screening recommendations (Supplementary Table 5, https://stacks.cdc.gov/view/cdc/133599). The median percentage of pregnant persons tested for HCV infection was 47.6% (range: 0.7%–98.4%) (Table 1). Thirty-five articles included data regarding anti-HCV positivity or hepatitis C diagnoses during pregnancy, 26 of which were reviewed in the 2020 CDC adult HCV screening recommendations. The median prevalence of anti-HCV positivity or diagnosis was 1.1% (range: 0.1%–70.8%). Eleven articles included data regarding HCV RNA positivity in pregnancy among those who were anti-HCV positive, of which four were reviewed in the 2020 CDC adult HCV screening recommendations. The median prevalence of HCV RNA positivity was 68.2% (range: 29.6%–81.3%).

Two articles presented data on the proportion of perinatally exposed children who were referred for HCV testing (Supplementary Table 6, https://stacks.cdc.gov/view/cdc/133599). The median percentage of children referred for testing was 16.7% (range: 1.9%–31.4%) (Table 1). Twelve articles presented data related to the proportion of perinatally exposed children tested for HCV infection, either with an anti-HCV test or NAT for HCV RNA. The median percentage tested for HCV infection was 30.1% (range: 8.6%–53.1%). Thirteen articles presented data regarding the rate of perinatal transmission. The median rate was 4.7% (range: 0.0%–11.1%). One article presented information regarding linkages to care among perinatally infected children and indicated that five of five (100%) were linked to care. Seven studies included data related to DAA treatment among perinatally HCV infected children aged 3–17 years (Supplementary Table 7, https://stacks.cdc.gov/view/cdc/133599). In these studies, the median percentage of children with chronic HCV infection who achieved sustained virologic response 12 weeks posttreatment was 98.1% (range: 96%–100%). Assessment of the quality of evidence was performed for each of the included articles using the NIH Study Quality Assessment tool, and the results ranged from fair to good (Supplementary Table 8, https://stacks.cdc.gov/view/cdc/133599).

Both U.S. and international articles were evaluated for harms associated with testing perinatally exposed children for HCV infection; six U.S. articles and 24 international articles were included (Supplementary Table 9, https://stacks.cdc.gov/view/cdc/133599). Among 30 studies that described potential harms, the most commonly reported harms were related to interpretation of test results, including intermittent or transient viremia (13 studies), false-positive antibody results (one study), and false-negative antibody results (two studies). Other harms included the cost of testing (four studies); stigma (four studies); guilt, stress, and concern about a child’s health, school, employment, and future marriage (one study); wait time for screening at 18 months (one study); misclassification of vertical transmission (one study); parental refusal of testing (one study); absence of approved treatment (one study); involvement of social services (one study); distance to follow up for families living far away (one study); time to go to the laboratory and wait for the test (one study); lack of testing and transportation availability (one study); pressure on clinic staff members to order and explain test results (one study); and delay in infection status or uncertain prognosis clarification contributing to parental confusion and poor clinic attendance (one study). All studies with harms were evaluated for the level of confidence and outcome prioritization (Supplementary Table 9, https://stacks.cdc.gov/view/cdc/133599). After careful evaluation and review, the Recommendations Steering Committee concluded that the benefits of testing outweighed the identified and potential harms of testing.

The data from the literature review are subject to at least four limitations. First, although data were abstracted in a consistent method, there was heterogeneity in the studies included (e.g., certain studies did not differentiate between the type of diagnostic test used [i.e., anti-HCV or NAT for RNA] or the age of the infant or child at the time the test was performed). In addition, studies might have defined maternal HCV infection as a single positive anti-HCV test, which might not represent current infection. Second, approximately 20 years of data and knowledge about perinatal HCV infection from 2001 to 2022 were included, including exposure and transmission, diagnosis, HCV RNA test performance, treatment, and outcomes. However, the data and knowledge about perinatal HCV infection have progressed substantially over this time. As a result, certain studies might have included previous definitions of HCV infection or less sensitive testing methods. However, because of the scarcity of data on perinatal HCV infection, including more rather than less data was essential. Third, with the exception of articles that described potential harms, articles with no U.S. data were excluded for all reviews to make the recommendations generalizable to the U.S. population on the basis of similar populations, medical care, treatment guidelines, and outcomes. However, this step might have excluded certain potentially relevant international studies. Finally, data from the systematic review of the literature were limited by the designs of the individual studies and the quality of the evidence. For this reason, standardized quality assessment tools created by NIH for observational cohort and cross-sectional studies and for before-after (prepost) studies with no control group (https://www.nhlbi.nih.gov/health-topics/study-quality-assessment-tools) were used to assess the quality of the evidence from included studies.

### Cost-Effectiveness Considerations

Evaluating the cost-effectiveness of public health interventions is critical to developing recommendations. No cost-effectiveness studies comparing testing approaches for perinatal HCV infections were identified during the development of these recommendations. Therefore, a CDC-conducted novel analysis evaluating the optimal testing strategy for perinatally exposed children guided these recommendations (*82*). Through CDC’s National Center for HIV, Viral Hepatitis, STD, and TB Prevention’s Epidemiologic and Economic Modeling Agreement, a mathematic modeling study was conducted using an economic analysis framework to compare the current strategy of anti-HCV testing with reflex to NAT for HCV RNA starting at age 18 months with a proposed strategy of a single NAT for HCV RNA at age 2–6 months. Also included were considerations for universal testing strategies for both options (i.e., all infants regardless of maternal HCV status). Inputs and estimates for the study were guided by published literature. For rate of hepatitis C screening during pregnancy, a mean estimate of 44.7% was used with sensitivity analyses to account for expected increases in screening during pregnancy because universal screening recommendations are becoming more widely implemented. In addition, decreased loss to follow-up was accounted for with the proposed strategy because more infants are expected to attend 2–6-month well-child visits than 18-month well-child visits (*83*). Costs and health outcomes of the various strategies were modeled and incorporated rates of spontaneous clearance of infection. Outcomes included diagnosed infections, treated or cured infections, HCC, liver transplants, and liver-related deaths.

The modeling study found that, compared with the baseline strategy of testing exposed children aged 18 months with an anti-HCV test and reflex to NAT for HCV RNA, an increased number of infants received diagnoses and had improved health outcomes when NAT for HCV RNA was performed at age 2–6 months among exposed children. Universal screening with both anti-HCV testing with reflex to NAT for HCV RNA at 18 months and NAT for HCV RNA at age 2–6 months also improved infant diagnoses and health outcomes. However, testing known exposed infants at age 2–6 months was the only strategy that was cost-saving compared with the baseline strategy, with a population level cost savings of $469,671 per year, assuming 3.6 million births with 0.64% of births occurring among persons with HCV infection. Although the universal testing strategies resulted in an increase in quality-adjusted life years (QALYs) compared with the baseline strategy, the strategies resulted in increased total costs ranging from $38 million for the universal anti-HCV test with reflex to NAT for the HCV RNA strategy at 18 months to $129 million for the universal NAT for HCV RNA at age 2–6 months (incremental cost-effectiveness ratios [ICERs] of 26,105 and 35,887 per QALY gained, respectively). Compared with NAT for HCV RNA among exposed children at age 2–6 months, universal NAT for HCV RNA screening becomes decreasingly cost-effective as the proportion of pregnant persons screened for HCV increases (i.e., because universal screening allows the HCV infection status of every pregnant person to be known, universal testing of infants would result in diminishing return). The strategy of testing known perinatally exposed infants with a NAT for HCV RNA at age 2–6 months was the only perinatal hepatitis C testing strategy that was both cost-saving and resulted in better health outcomes. Limitations of the study included a paucity of data on testing strategies for infants and children exposed to perinatal HCV, attributing the cost and benefits of testing only to the exposed child, assuming perinatal HCV not diagnosed in childhood would not be diagnosed and treated later in life and assuming that there was no difference in linkage to treatment or costs before age 3 years with the different testing strategies. A health economist determined the study met the overall standards for quality of the CHEERS checklist (Supplementary Table 10, https://stacks.cdc.gov/view/cdc/133599). The steering committee determined that any minor deviations from the checklist were appropriate and did not compromise the quality of the evidence.

### Rationale for Recommendations

Rates of HCV infections during pregnancy have been increasing (*6*,*7*), corresponding with the ongoing opioid crisis (*11*). Although perinatal HCV transmission occurs in up to 7% of perinatally exposed children (*13*,*14*), approximately 70% of children aged ≥18 months are not being tested with the current strategy of anti-HCV testing, leading to loss to follow-up (Table 1). With the availability of highly sensitive and specific NATs for HCV RNA detection starting at age 2 months (*79*) and more children attending well-child visits at age 2–6 months compared with those aged ≥18 months (*17*,*84*), a NAT for HCV RNA at age 2–6 months is both cost-effective and cost-saving and will identify more children with perinatal HCV transmission who are eligible for curative treatment beginning at age 3 years (*82*).

### HCV Testing Strategy

HCV testing of children exposed perinatally identifies children who are at risk for developing complications from chronic HCV infection. Standard clinical practice for testing with a NAT for HCV RNA is done at or after age 2 months, and testing with anti-HCV is done at or after age 18 months because of the persistence of antibody passively transferred from the infected birthing parent to the infant (*37*,*85*–*87*). The older, less sensitive NAT for HCV RNA used in certain studies of perinatally exposed infants was associated with potential false-negative results indicating intermittent viremia (*37*,*88*,*89*); however, currently used tests are highly sensitive with a lower limit of detection of 15.0 IU/mL or less for genotype 1a (*90*). One NAT for HCV RNA at age 2–6 months is sufficient to determine perinatal infection. On the basis of data related to the sensitivity, specificity, and PPVs and NPVs, a detectable HCV RNA result confirms perinatal HCV transmission and an undetectable HCV RNA rules out perinatal HCV transmission (*79*,*85*,*88*,*91*). If anti-HCV testing is done at or after age 18 months, a NAT for HCV RNA on all reactive specimens will identify current HCV infection. Children with nonreactive anti-HCV tests at age ≥18 months and children with undetectable HCV RNA test results after a reactive anti-HCV test at age ≥18 months do not need further follow-up.

## CDC Recommendations for Hepatitis C Testing Among Perinatally Exposed Infants and Children

CDC recommends HCV testing for all infants and children born to pregnant persons with current or probable HCV infection (Box). A pregnant person with a current HCV infection has detectable HCV RNA. A pregnant person is considered to have a probable infection if anti-HCV testing is reactive and HCV RNA results are not available.

BOX **CDC recommendations for hepatitis C testing among perinatally exposed infants and children — United States, 2023**

- All infants and children born to pregnant persons with current or probable hepatitis C virus infection should be tested for hepatitis C.
  - Pregnant persons with detectable HCV RNA are considered to have current HCV infection. If anti-HCV testing is reactive and HCV RNA results are not available, pregnant persons are considered to have probable HCV infection.*
- Perinatally exposed infants should receive a NAT for HCV RNA at age 2–6 months to identify children in whom chronic HCV infection might develop.^†^
  - Infants with detectable HCV RNA should be managed in consultation with a health care provider with expertise in pediatric hepatitis C management.
  - Infants with undetectable HCV RNA do not require further follow-up.^§^
- Other considerations
  - Infants and children aged 7–17 months who have not previously been tested should receive a NAT for HCV RNA.
  - Children aged ≥18 months who previously have not been tested should receive an anti-HCV test with reflex^¶^ to NAT for HCV RNA.

**Abbreviations:** anti-HCV = hepatitis C virus antibody; FDA = Food and Drug Administration; HCV = hepatitis C virus; NAT = nucleic acid test.

* Because a pregnant person with a detectable HCV RNA test anytime during pregnancy (even if followed by an undetectable HCV RNA test) can potentially transmit HCV during the infectious period, the infant should be tested for HCV RNA. If no HCV test is available from pregnancy and the most recent NAT indicates detectable HCV RNA in the absence of treatment, a pregnant person is considered to have current HCV infection. Similarly, if no HCV test is available from pregnancy and the most recent anti-HCV test is reactive in the absence of an undetectable HCV RNA or treatment, a pregnant person is considered to have probable HCV infection.

^†^ Off-label use of an FDA-approved diagnostic test requires validation by the testing laboratory.

^§^ No further follow-up needed unless clinically warranted (e.g., clinical symptoms, signs, or laboratory findings consistent with hepatitis C).

^¶^ A NAT for HCV RNA performed on specimens that are anti-HCV reactive.

### Testing

**FIGURE 3:**
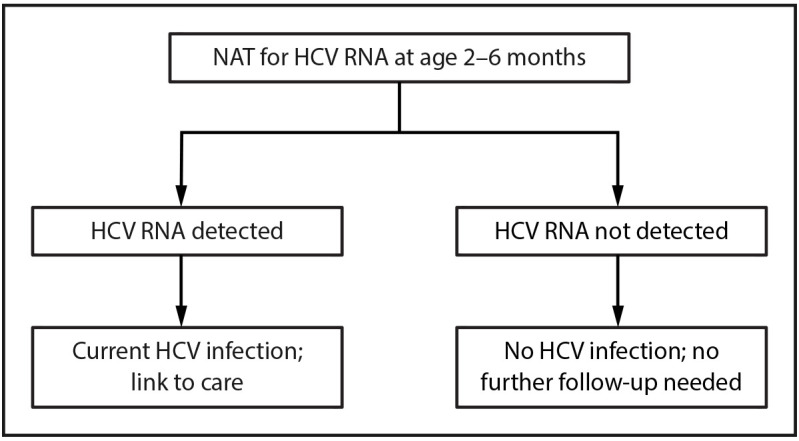
Algorithm for hepatitis C virus testing of perinatally exposed children — United States, 2023*^,†,§,¶^ **Abbreviations:** FDA = Food and Drug Administration; HCV = hepatitis C virus; NAT = nucleic acid test. * Perinatally exposed children are children born to pregnant persons with HCV infection. ^†^ Perinatally exposed children aged 7–17 months who have not previously been tested also should receive a NAT for HCV RNA. ^§^ Off-label use of an FDA-approved diagnostic test requires validation by the testing laboratory. ^¶^ No further follow-up needed after a negative HCV RNA performed at age 2–6 months unless clinically warranted (i.e., clinical symptoms or signs or laboratory findings consistent with hepatitis C).
  - Infants with detectable HCV RNA should be managed in consultation with a health care provider with expertise in pediatric hepatitis C management.
  - Infants with an undetectable HCV RNA result do not require further follow-up unless clinically warranted.
- Infants and children aged 7–17 months who are perinatally exposed to HCV and have not previously been tested should receive a NAT for HCV RNA.
- Children aged ≥18 months who are perinatally exposed to HCV and have not previously been tested should receive an anti-HCV test with reflex to NAT for HCV RNA (Figure 4).

- Perinatally exposed infants should receive a NAT for HCV RNA at age 2–6 months to identify children in whom chronic HCV infection might develop if not treated (Figure 3).^†^

**FIGURE 4:**
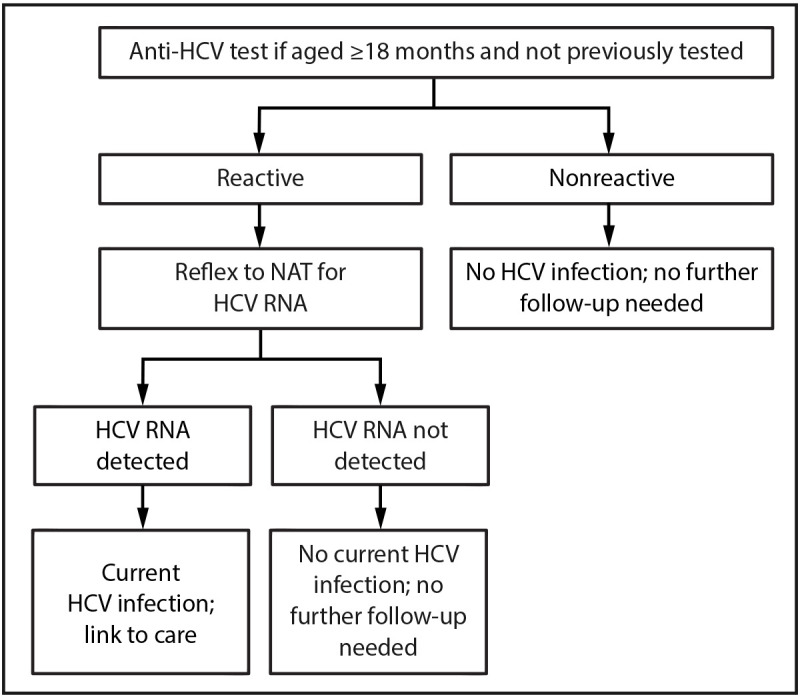
Algorithm for hepatitis C virus testing of perinatally exposed children* aged ≥18 months who have not previously been tested^†^ — United States, 2023 **Abbreviations:** anti-HCV = hepatitis C virus antibody; HCV = hepatitis C virus; NAT = nucleic acid test. * Perinatally exposed children are children born to pregnant persons with HCV infection. ^†^ Not tested for perinatal HCV transmission with a NAT for HCV RNA at age 2–17 months.

#### Evidence Summary

Rates of hepatitis C among reproductive-aged persons have been increasing (*4*), resulting in increasing rates of hepatitis C during pregnancy (*6*,*7*), but systematic review of data from 12 studies illustrate that only 30.1% of perinatally exposed infants and children are tested for HCV infection (Table 1). With perinatal transmission rates of up to 7% among exposed infants (*13*,*14*), the majority of children with current infection remain undiagnosed and are lost to follow up. One surveillance study indicated only 16% of exposed infants were tested for HCV infection, leaving most children with current HCV infection unidentified (*20*). The most common harms related to testing for perinatal HCV infection relate to interpretation of test results, including intermittent or transient viremia, false-positive antibody results, false-negative antibody results, the cost of testing, and stigma. However, currently used NATs for HCV RNA tests are highly sensitive and specific for diagnosing perinatal HCV transmission (*79*). Furthermore, more children attend well-child visits and receive perinatal HCV testing during the first 6 months of life compared with well-child visits at 18 months (*17*,*84*). Finally, early diagnosis of perinatal HCV transmission at age 2–6 months was determined to be cost-effective and cost-saving in preventing morbidity and mortality from chronic hepatitis C complications (*82*); curative treatment is available starting at age 3 years. As a result, the benefits of testing were determined to outweigh any potential and identified harms.

### Clinical Considerations for Children with Unknown HCV Exposure During Pregnancy

The risks for and benefits of testing children born to pregnant persons with unknown hepatitis C status were not directly assessed. Evidence indicates that siblings of children with perinatal hepatitis C exposure born to the same birth parent would benefit from being tested for HCV infection unless the birth parent was known to be HCV negative (i.e., HCV RNA not detected) during the previous pregnancy (*15*,*16*). Testing can be performed starting at age 2 months with a NAT for HCV RNA, or at age ≥18 months with an anti-HCV test with reflex to NAT for HCV RNA. In addition, children whose birth parent’s hepatitis C status is unknown because the child and birth parent are separated (e.g., children in foster care or infants safely surrendered after birth) or other situations where the birth parent cannot be tested also would benefit from being tested starting at age 2 months with a NAT for HCV RNA or at age ≥18 months with an anti-HCV test with reflex to NAT for HCV RNA (*84*,*92*–*94*). If a pregnant person was at risk for having an acute infection during pregnancy (e.g., IDU) and was not tested close to the time of delivery, the infant also would benefit from being tested.

### Patient Follow-Up

Standard practice calls for infants and children with perinatally acquired HCV infection (detectable HCV RNA at or after age 2 months) to be managed in consultation with a provider with expertise in pediatric hepatitis C management to receive related screenings, preventive services, interventions, and regular follow-up. To confirm chronic hepatitis C, children who test positive should be retested with a NAT for HCV RNA before beginning treatment, which can be started as early as age 3 years. Detailed management guidelines from AASLD-IDSA are available at https://www.hcvguidelines.org/unique-populations/children.

- Parents or guardians of perinatally exposed children with undetectable HCV RNA aged ≥2 months can be reassured that the child does not have perinatal HCV infection and does not require further follow-up. If clinical symptoms, signs, or laboratory findings consistent with hepatitis C appear later in childhood, retesting is reasonable because rare false-negative test results and postnatal acquisition of the infection through other means are possible.
- Parents or guardians of perinatally exposed children aged ≥18 months with nonreactive anti-HCV test results can be reassured that the child does not have perinatal HCV infection and does not require further follow-up.
- Parents or guardians of perinatally exposed children aged ≥18 months with reactive anti-HCV test results and undetectable HCV RNA can be reassured that although there was likely perinatal HCV transmission, the child does not have current HCV infection and does not require further follow-up. A reactive antibody at age 18 months with an undetectable HCV RNA also might represent presence of maternal antibody (*37*).

### Reporting

Cases of perinatal hepatitis C should be reported to the appropriate state or local health department in accordance with requirements for reporting perinatal HCV infections. Case definitions for reportable cases have been published by the Council of State and Territorial Epidemiologists (*23*).

### Perinatal HCV Testing Recommendations of Other Organizations

CDC testing recommendations differ in certain ways from those of other organizations, particularly on the ages at which testing should occur (Table 2). CDC testing recommendations focus on an earlier infant age range of 2–6 months because of increasing rates of pediatric hepatitis C, high rates of loss to follow-up among exposed infants and children, and the increased effectiveness and cost-effectiveness of earlier hepatitis C diagnosis with a NAT for HCV RNA in young infants. As of 2023, AASLD-IDSA (*15*), NASPGHAN (*16*), and AAP (*78*) recommend anti-HCV testing at age ≥18 months with reflex to NAT for HCV RNA among perinatally exposed children. These organizations also recommend considering a NAT for HCV RNA at age ≥2 months in certain circumstances. AAFP recommends two separate NATs for HCV RNA at age 2–6 months or an anti-HCV test at age ≥15 months (*3*). AASLD-IDSA and AAP recommend an anti-HCV test ≥18 months, regardless of the exposed child’s HCV RNA result during infancy. AASLD-IDSA also recommends a NAT for HCV RNA at age 3 years for those with perinatal HCV transmission to confirm chronic infection. AASLD-IDSA and NASPGHAN recommend screening siblings of perinatally infected children.

**TABLE 2:** Perinatal HCV testing recommendations, by organization — United States, 2023

Organization	NAT for HCV RNA at age ≥2–6 months?	Confirm anti-HCV at age ≥18 months?	Anti-HCV with reflex* NAT for RNA at age ≥18 months?	Retest for HCV RNA before initiating treatment?	Test siblings?
**CDC (2023)**	Yes^†^	No	If not previously tested	Yes	Yes
**AAP (2021 Red Book)** ^§^	Consider	Yes	Yes	NA	NA
**AASLD-IDSA (2020)** ^¶^	Consider**	Yes	Yes	Yes	Yes
**NASPGHAN (2020)** ^††^	Consider^§§^	NA	Yes	Yes	Yes
**AAFP (2010)** ^¶¶^	Yes^***^	NA	Yes	NA	NA

**Abbreviations:** AAFP = American Association of Family Physicians; AAP = American Academy of Pediatrics; AASLD = American Association for the Study of Liver Diseases; anti-HCV = hepatitis C virus antibody; HCV = hepatitis C virus; ISDA = Infectious Diseases Society of America; NASPGHAN = North American Society for Pediatric Gastroenterology, Hepatology, and Nutrition; NA = not available; NAT = nucleic acid test; NIH = National Institutes of Health.

* NAT for HCV RNA performed on specimens that are anti-HCV reactive.

^†^ NAT for HCV RNA can be done at age ≤17 months; at age ≥18 months, recommend anti-HCV testing with reflex to NAT for HCV RNA if not previously tested.

^§^ **Source:** Kimberlin DW, Barnett ED, Lynfield R, Sawyer MH, editors. Red Book 2021–2024: report of the Committee on Infectious Diseases, 32nd edition. Itasca, IL: American Academy of Pediatrics; 2021.

^¶^ **Source:** Infectious Diseases Society of America. HCV in children. Arlington, VA: Infectious Diseases Society of America; 2022. Accessed June 2, 2022. https://www.hcvguidelines.org/unique-populations/children

** Consider at age 2–12 months.

^††^ **Source:** Leung DH, Squires JE, Jhaveri R, et al. Hepatitis C in 2020: a North American Society for Pediatric Gastroenterology, Hepatology, and Nutrition position paper. J Pediatr Gastroenterol Nutr 2020;71:407–17.

^§§^ Consider at age 2 months if requested by family; recheck at age 12 months to confirm chronic hepatitis C.

^¶¶^ **Source:** Wilkins T, Malcolm JK, Raina D, Schade RR. Hepatitis C: diagnosis and treatment. Am Fam Physician 2010;81:1351–7.

*** AAFP recommendations based on a NIH consensus statement (now retired). These guidelines recommend a NAT for HCV RNA on two separate occasions between age 2–6 months or anti-HCV testing at age ≥15 months.

## Future Directions

CDC will continue to monitor changes in epidemiology, diagnosis, and treatment of hepatitis C during pregnancy and among children, and future revisions to these recommendations might be indicated if new and substantial evidence becomes available. If DAA treatments are approved for pregnant persons and widely used, the resulting clearance of maternal viremia during pregnancy will lead to fewer children becoming infected. Although these recommendations included a systematic review of approximately 20 years of literature during 2001–2022 on hepatitis C in pregnancy and children exposed perinatally, multiple gaps in the literature exist. More data are needed to understand the implications of universal screening during pregnancy and the true prevalence of hepatitis C among pregnant persons. Furthermore, data on the prevalence and natural history of perinatal hepatitis C are needed because limited prospective longitudinal studies following a cohort of children infected perinatally and evaluating outcomes exist. Better understanding is needed regarding how to improve linkage to care for these infants, specifically barriers to testing and reasons why testing is not done and identified children are not referred to appropriate providers for follow-up. Surveillance for children infected perinatally before and after these recommendations also will guide further understanding of progress toward hepatitis C elimination, specifically whether an earlier diagnosis of perinatal hepatitis C is associated with higher treatment and cure rates. Jurisdictional viral hepatitis programs can improve surveillance, diagnosis, and linkage to care if provided with additional funding and resources for capacity building and implementation to expand existing viral hepatitis infrastructure. Finally, resources to educate pediatric providers on the importance of testing and treating children for hepatitis C, including financial reimbursement, awareness of stigma, approaches to counseling patients regarding the implications of a positive test, and information on the safety of breastfeeding, will be needed as more pregnant persons and exposed children are identified through testing.

## Conclusion

Using a testing strategy of highly sensitive and specific NATs for RNA detection among infants and children perinatally exposed to HCV increases the identification of children with HCV infection in whom substantial morbidity and mortality might develop. HCV infection will develop in approximately 6%–7% of all perinatally exposed infants and children (*13*,*14*), and curative DAA therapy can be administered starting at age 3 years (*15*,*16*). However, most perinatally exposed children are not tested for HCV infection and are not referred for hepatitis C care (*17*–*20*). Reasons for this might include lack of awareness of perinatal exposure by pediatric providers, lack of regular pediatric care among exposed children, and changes in health care providers before the time of HCV testing (currently recommended at age 18 months). Testing perinatally exposed infants beginning at age 2 months with a NAT for HCV RNA is cost-effective and allows for earlier linkage to care, appropriate evaluation, and the opportunity to provide curative, life-saving therapy. The identification of and linkage to curative treatment for all persons with HCV infection (including infants and children) is essential to ensuring that no population is left behind in the pursuit of national hepatitis C elimination goals. Because more pregnant persons with HCV infection are identified through universal screening, more infants and children with identified exposure will seek care at various stages. Regardless of when a child is seen by a provider, opportunities exist for education, testing and evaluation, curative treatment, and progress toward the goal of hepatitis C elimination (*95*).

## Footnotes

* The comparator of infants aged 2–6 months was chosen because the period is the earliest when a perinatally exposed infant can be tested and when children are more likely to be engaged in routine care. https://publications.aap.org/pediatrics/article/142/5/e20174019/38551/Gaps-in-Well-Child-Care-Attendance-Among-Primary?autologincheck=redirected

† Off-label use of an FDA-approved diagnostic test requires validation by the testing laboratory.
